# Laser heating system at the Extreme Conditions Beamline, P02.2, PETRA III

**DOI:** 10.1107/S1600577521009231

**Published:** 2021-10-07

**Authors:** Zuzana Konôpková, Wolfgang Morgenroth, Rachel Husband, Nico Giordano, Anna Pakhomova, Olof Gutowski, Mario Wendt, Konstantin Glazyrin, Anita Ehnes, Jan Torben Delitz, Alexander F. Goncharov, Vitali B. Prakapenka, Hanns-Peter Liermann

**Affiliations:** a Deutsches Elektronen-Synchrotron (DESY), Notkestrasse 85, 22607 Hamburg, Germany; b European XFEL GmbH, Holzkoppel 4, Schenefeld, Germany; c Institut für Geowissenschaften, Kristallographie/Mineralogie, Goethe Universität Frankfurt am Main, Altenhöferallee 1, D-60438 Frankfurt am Main, Germany; d Earth and Planets Laboratory, Carnegie Institution for Science, 5251 Broad Branch Rd NW, Washington, DC 20015, USA; e Center for Advanced Radiation Sources, University of Chicago, Chicago, IL 60637, USA

**Keywords:** pulse laser heating, ray-tracing simulations, extreme conditions, diamond anvil cell, high-pressure melting of iron

## Abstract

A laser heating system for samples confined in diamond anvil cells paired with *in situ* X-ray diffraction measurements at the Extreme Conditions Beamline of PETRA III is presented. The system features two independent laser configurations (on-axis and off-axis of the X-ray path) allowing for a broad range of experiments using different designs of diamond anvil cells. The power of the continuous laser source can be modulated for use in various pulsed laser heating or flash heating applications. An example of such an application is illustrated here on the melting curve of iron at megabar pressures. The optical path of the spectroradiometry measurements is simulated with ray-tracing methods in order to assess the level of present aberrations in the system and the results are compared with other systems, that are using simpler lens optics. Based on the ray-tracing the choice of the first achromatic lens and other aspects for accurate temperature measurements are evaluated.

## Abstract 2

A laser heating system for samples confined in diamond anvil cells paired with *in situ* X-ray diffraction measurements at the Extreme Conditions Beamline of PETRA III is presented.

## 1. Introduction

For over more than half a century diamond anvil cells (DACs) have been used to simulate high static pressure conditions to study properties of materials under extreme conditions encountered in astro- and planetary physics (Sanloup *et al.*, 2013 ▸) or to study synthesized compounds in material chemistry (Zhang *et al.*, 2013 ▸) and physics (Scheler *et al.*, 2013 ▸). Introducing heat to the sample has been accomplished by using either resistive heating techniques for temperatures up to 1200–1500 K [recently temperatures beyond 5000 K were reported (Sinmyo *et al.*, 2019 ▸)] or laser heating up to temperatures of 1000–10000 K (Goncharov *et al.*, 2009 ▸). Probing the properties of the materials *in situ* at these extreme states has been very successful at third-generation light sources that offer high energy, low emittance and thus highly brilliant X-ray beams. For this reason most synchrotron storage rings, but in particular the large third-generation light sources, have at least one beamline station that is dedicated to the study of extreme conditions in the laser heated DAC (Yagi *et al.*, 2001 ▸; Schultz *et al.*, 2005 ▸; Meng *et al.*, 2006 ▸; Prakapenka *et al.*, 2008 ▸; Boehler *et al.*, 2009 ▸; Kupenko *et al.*, 2012 ▸; Fukui *et al.*, 2013 ▸; Petitgirard *et al.*, 2014 ▸; Meng *et al.*, 2015 ▸; Aprilis *et al.*, 2017 ▸; Stan *et al.*, 2018 ▸; Kantor *et al.*, 2018 ▸; Anzellini *et al.*, 2018 ▸; Fedotenko *et al.*, 2019 ▸; Spiekermann *et al.*, 2020 ▸).

The principle of laser heating systems for DACs is very similar at the different high-pressure beamlines. They consist of the heating laser and an optical path to view the sample and measure the temperature by spectral radiometry. However, they differ in small details such as the angle between the incident heating laser beams and the X-ray beam, sample visualization or choice of the light-collecting optics. Typically, near-infrared (NIR) (∼1060 ± 10 nm) laser light is used for heating samples with high absorbance at these wavelengths. The laser beam is applied on both sides of the sample to minimize the axial temperature gradients. Transparent samples, however, need to be mixed with a ‘coupler’, an opaque powder or foil, in order to absorb the NIR radiation. Mixing samples with couplers is not always desirable due to possible chemical reactions. Alternatively, CO_2_ lasers, with longer wavelength of 10.6 µm, are commonly employed to heat transparent samples (Smith *et al.*, 2018 ▸; Petitgirard *et al.*, 2014 ▸). The difficulty with CO_2_ laser heating is the necessity to have separate optics for the laser and the radiometry/visualization paths, difficult alignment procedures and the fact that the minimum focal spot size at such long wavelengths (∼50 µm) is not compatible with experiments at multimegabar pressures due to the small sample size.

Initially, heating using lasers in continuous-wave (cw) mode was the standard method of heating the samples in DACs. Recently, pulsed laser heating has been recognized to be beneficial in certain experiments (Goncharov *et al.*, 2010 ▸; McWilliams *et al.*, 2015*a* ▸,*b* ▸). With laser pulses one may reach higher temperatures by avoiding overheating of the entire DAC assembly, and higher stability of the heating by minimizing the ‘run-away’ effects (sudden uncontrollable increase in temperature) due to, for example, phase transitions, chemical reactions or metallization (McWilliams *et al.*, 2015*a* ▸). Tracking a time-resolved response of the sample to the heating pulses can also yield information on the transient properties of the sample such as thermal and electrical conductivities (Konôpková *et al.*, 2016 ▸; Beck *et al.*, 2007 ▸; Goncharov *et al.*, 2009 ▸).

Therefore, additional to the cw heating, pulsed laser heating in the DAC has been developed at the Extreme Conditions Beamline, PETRA III, for time-resolved measurements, single pulsed-heating or pump-and-probe experiments. These developments have been enabled by high brilliance at high photon energies of the third-generation light sources and modern fast large-area gated X-ray detectors. While a detailed description of the Extreme Conditions Beamline is presented elsewhere (Liermann *et al.*, 2015 ▸), we focus here on the laser heating setup in more detail. Analysis of the optical aberrations is presented using the ray-tracing program *ZEMAX*. Different pulsed laser heating methods are described, which are then applied to the melting behavior of iron at pressures up to 2 Mbar.

### 1.1. Extreme Conditions Beamline, P02.2, PETRA III

The Extreme Conditions Beamline (ECB) (Liermann *et al.*, 2015 ▸) is dedicated to research on matter at static and dynamic high pressures using primarily the DAC technique. The main X-ray techniques at simultaneously high pressure and low/high temperatures are powder and single-crystal diffraction and scattering from non-crystalline materials. In order to penetrate ∼6 mm of diamond material when using DACs in transmission geometry, it is desirable to use high-energy X-rays. The ECB operates mostly at fixed energies of 25.6 keV and 42.7 keV. Two types of focusing are available – compound refractive lenses (CRLs) and the Kirkpatrick–Baez (KB) mirror system. The minimum spot size achievable using the CRLs is 8 µm (horizontal) × 2 µm (vertical) FWHM while the KB mirrors can produce a focus of 1.5 µm × 1.5 µm. ECB features two experimental setups with identical X-ray focusing capabilities. Situated upstream is the general purpose (GP) experiment for sample environments such as cryostats, piezo-driven dynamic or graphite resistive-heated DACs. Located downstream from the GP experiment is the laser-heating (LH) table offering permanent installations of near-infrared YAG and CO_2_ lasers, optics for temperature measurements and ruby system for online pressure measurements.

### 1.2. Laser heating at ECB

**Figure 1:**
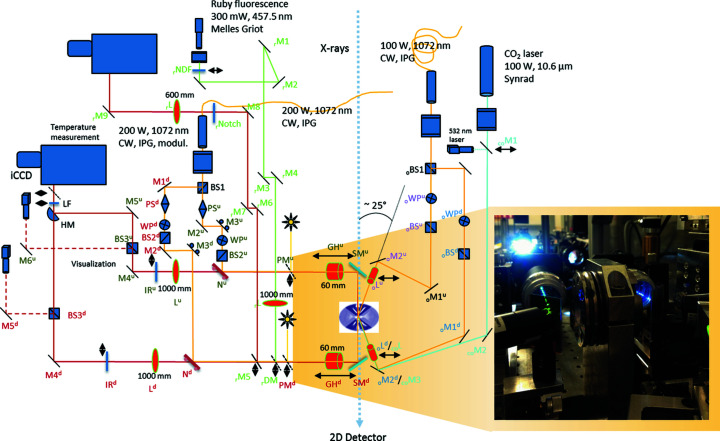
Sketch of the laser heating systems at the Extreme Conditions Beamline at PETRA III at DESY, Hamburg. Superscripts ‘d’ and ‘u’ refer to ‘downstream’ and ‘upstream’, respectively. Subscript ‘r’ denotes the ruby fluorescence part, ‘o’ the off-axis path and ‘co’ the CO_2_ laser path. SM – Semrock mirror, GH – geoHEAT, DM – dichroic mirror for 457 nm, N – dichroic mirror for 1072 nm, L – lens, IR – laser filter, M – mirror, BS – beamsplitter, WP – waveplate, PS – πShaper.

**Figure 2:**
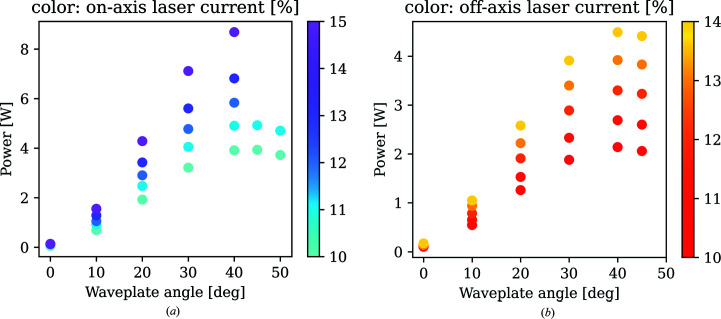
Measured laser power as a function of waveplate angle (*x*-axis) and laser current (color bar, in %) for one beam of the (*a*) on-axis laser (100 W maximum power after splitting) and (*b*) off-axis laser (50 W maximum power after splitting).

Two cw (1072 nm) ytterbium fiber lasers are used for two independent double-side laser heating systems, called *on-axis* and *off-axis* (axis being defined by the X-ray beam), in order to accommodate any DAC seat openings in order to increase flexibility of the laser system geometry (Fig. 1 ▸). For the *off-axis* setup, one 100 W laser (IPG Laser GmbH, YLR-100-SM, 5 mm beam diameter) is split with a polarizing beamsplitter (_o_BS1) and guided to the sample at an angle of about 25° to the X-ray beam (*i.e.* off-axis) and is thus de-coupled from the observation/temperature measurement part. The second, 200 W laser (IPG Laser GmbH, YLR-200-AC, 5 mm beam diameter), powering the *on-axis* setup, is also split but inserted into the observation path using a dichroic mirror (N) and hence propagating to the sample co-axially with the X-ray beam (*i.e.* on-axis). All laser beam paths include a combination of polarizing cube beamsplitters (BS2 in *on-axis* and _o_BS in *off-axis*) and a waveplate (λ/2, Edmund Optics, 1064 nm, WP and _o_WP) for individual fine power tuning of all laser beams. The laser power (in W) as a function of the nominal laser output and waveplate rotation was measured at the sample position for the upstream beams of the on-axis and off-axis laser beam paths as a reference for the users [Figs. 2 ▸(*a*) and 2(*b*)].

The laser focusing optics in the two systems is different: the laser in the *off-axis* system is focused onto the sample by simple plano-convex, 1/2 inch lenses with an anti-reflection coating at 1064 nm and an effective focal length of 50.2 mm. The typical laser spot size is a Gaussian shape of 20 µm (FWHM) (measured at best focus with Thorlabs optical beam profiler). In the *off-axis* system, the laser part is de-coupled from the temperature measurement part, and hence it is possible to de-focus the beam by moving the lenses along their optical axis to achieve a larger heating area. In the *on-axis* system, the laser is coupled to the temperature measurement beam path, and therefore a special objective is used for both focusing of the laser beam and collecting of the thermal radiation. The objective (geoHEAT-60-NIR, AdlOptica) is optimized on a diffraction-limited level for both optical paths; in the visible range (600–900 nm) and for the laser wavelength (1020–1100 nm). Both legs of the 200 W laser after the splitting travel through beam shapers (F-πShaper 9-1064, AdlOptica) for independent shaping of the focal spot.

The transfer function of the optical system for temperature measurement is determined using a tungsten halogen lamp (OPTEEMA Engineering GmbH, OL-245M-K3) calibrated for three different temperatures: 2200 K, 2500 K and 2900 K. The lamp’s wire is imaged onto the CCD camera and the reference spectra are taken from upstream and downstream separately. The spectroradiometric measurements are typically performed in the spectral range 640–850 nm. In addition to a notch filter blocking the YAG laser line a longpass filter with a cut-off below 640 nm is placed in front of the spectrograph to eliminate the contribution from the second-order harmonics of lower wavelengths. Spectra from the hot sample, background corrected, are then used for temperature calculation by fitting to the Planck’s law using, for example, a software T-Rax by C. Prescher (github.com/CPrescher/T-Rax). The system for off-axis CO_2_ laser heating will be described elsewhere.

### 1.3. X-ray beam/laser beams alignment

**Figure 3:**
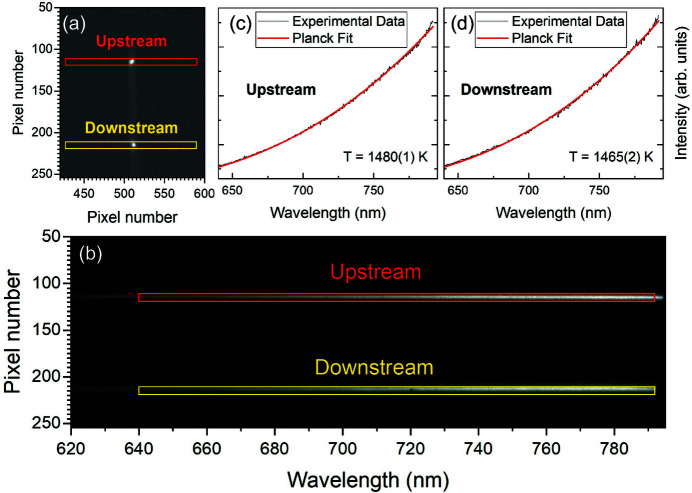
(*a*) Image of a 5 µm pinhole at the sample position collected on the Andor iStar camera with the spectrometer in the imaging mode, which is used to co-align the spectrometer and microscope cameras. The red and yellow boxes indicate the vertical pixel range that should be used for the ROI for temperature measurements. (*b*)–(*d*) Examples of pyrometry data collected from a 2 µm Pt foil in a DAC which is heated in cw mode on-axis. The software used in this example is *T-Rax* (Prescher & Prakapenka, 2015 ▸).

The optical beam-paths (upstream and downstream) are split after the second focusing lens (*f* = 1 m) by a 50–50 cube beamsplitter (BS3 in Fig. 1 ▸) into two legs; in one, light is focused onto the spectrometer slit; the second forms an optical microscope with a CCD camera (Allied Vision) for *in situ* observation of the sample. The field of view of the microscope is fixed and approximately 300 µm. An image of a back-illuminated 5 µm pinhole placed at the sample position is used to co-align the imaging camera and the spectrometer camera. While the image of the pinhole is positioned on a reference mark in the center of the field of view of the microscope camera, the pinhole can simultaneously be imaged on the spectrometer camera when the spectrometer is in imaging mode [Fig. 3 ▸(*a*)]. The downstream and upstream optical paths are merged before the spectrometer entrance by a D-shaped (half) mirror which reflects the upstream part while the downstream path propagates over the mirror. The two optical paths are thus vertically offset. This allows for two regions of interest (ROIs) to be identified on the spectrometer CCD/ICCD for upstream and downstream temperature measurement, respectively [Fig. 3 ▸(*b*)]. The ROI selection using the 5 µm pinhole is illustrated for the iStar camera in Fig. 3 ▸, and example thermal spectra are shown for a sample of Pt foil in a DAC. To ensure that temperature is collected from the region of the sample that is probed by the X-rays, the X-ray position must therefore be precisely aligned to the reference mark by adjusting the first mirror and objective of the laser heating optical system (SM in Fig. 1 ▸). The X-ray position is usually possible to directly visualize by the X-ray induced fluorescence of the sample using high exposure time and high gain settings of the optical microscope.

Once the X-ray spot and the radiometry path (and observation) are aligned, all laser beams are brought to the same spot by tweaking a dichroic mirror (in the *on-axis* system, N in Fig. 1 ▸) or a mirror/lens system (in the *off-axis* system, _o_L and _o_M2). Visualization and illumination of the sample in transmitted and reflected LED light is possible also during laser heating by inserting thin pellicle beamsplitters (PM in Fig. 1 ▸).

The positioning of the image of the sample, the laser and X-ray beams to the reference mark from both sides of the DAC secures the alignment with respect to the spectrometer–detector system throughout the experiment.

### 1.4. Temperature measurement, choice of spectral range

Light emitted from the hot sample is collected from both sides by a system of mirrors and lenses and guided to the spectrometer entrance slit. The first mirror, which reflects the beam by 90°, is a flat, thin mirror optimized for imaging with high reflectivity (Semrock, MGP01-650–1300) at the laser wavelength [reflection > 98%, 650–1300 nm; reflection (s-pol) > 99.5%, 650–1300 nm, reflection (p-pol) > 96%, 650–1300 nm]. The mirror is only 1.1 mm thick, causing little hard X-ray attenuation. A small lead beamstop is mounted on the back side of the downstream mirror to absorb the incident X-ray beam and scattering from the downstream mirror. The diverging radiation originating from the hotspot in the sample is collected by the geoHEAT 60-NIR, designed to compensate aberrations when used in combination with a BK7 lens of 750 mm focal length (in the current setup it is paired with an *f* = 1000 mm lens made of BK7 glass). Thermal spectra are recorded using a Shamrock spectrometer (SR-303i-A-SIL) equipped with two detectors: an iStar ICCD (DH320T-18U-73) and an Andor iDUS CCD (DU420A-BEX2-DD) that is used in conjunction with a shutter installed in the spectrometer, where switching between the two detectors is possible using an internal flipping mirror. The iStar detector is an intensified gated camera with a shortest gating time of 2 ns and is used for time-resolved measurements, whereas the iDUS is used for cw measurements at low temperatures due to its higher quantum efficiency (>90% in the 600–850 nm region) than the iStar camera (<30%). The sensor pixel size is 26 µm for both the iStar and iDUS camera. The optical magnification of the system is 16.6, hence 5 µm in the object plane corresponds to about 3 pixels of the camera sensor.

**Figure 4:**
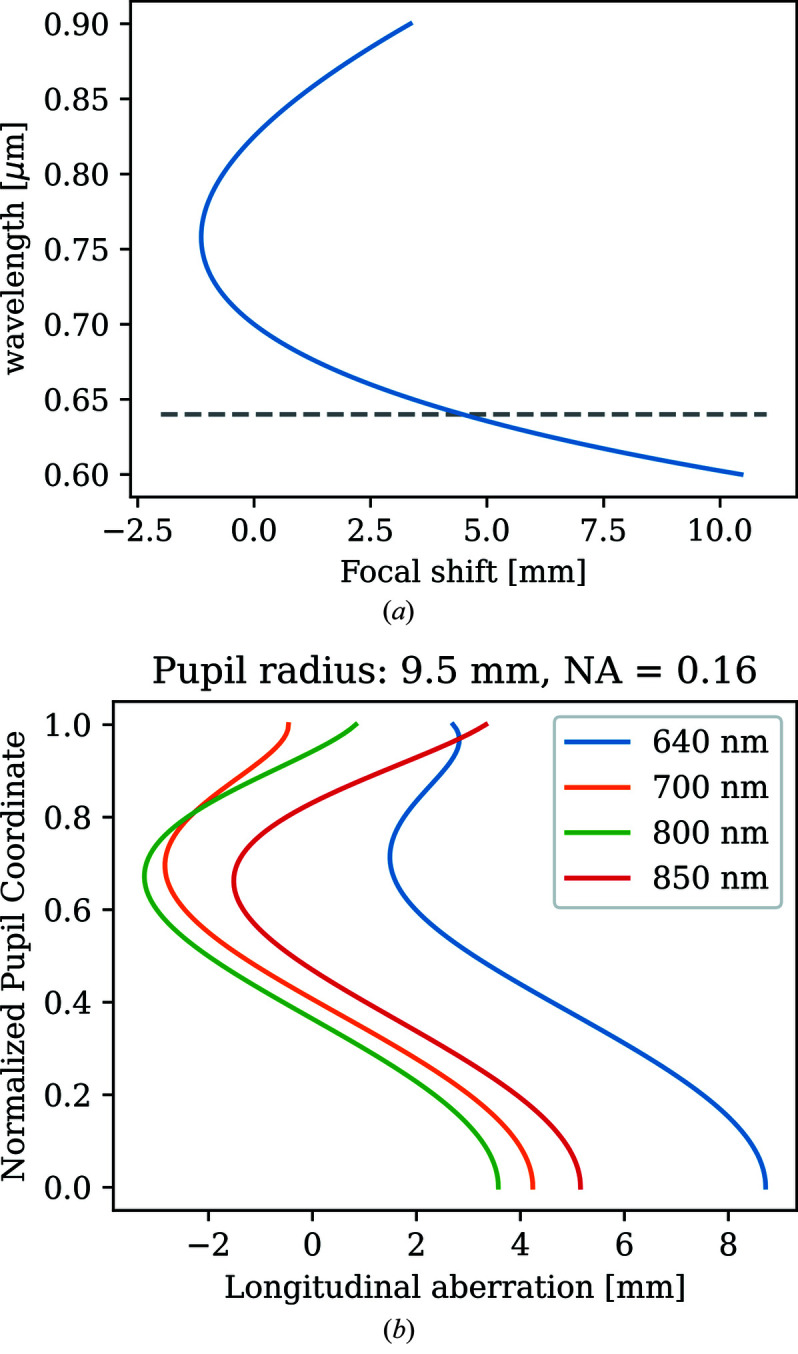
(*a*) Chromatic focal shift in the wavelength range 600–900 nm, for geoHEAT lens and *f* = 1 m BK7 lens, paraxial rays. The dashed line shows the cut-off wavelength above which the chromatic aberrations are significantly reduced. (*b*) Longitudinal aberration for 640–850 nm along the whole lens aperture (19 mm diameter).

A very thorough discussion on the accuracy of temperature measurements from DACs is given by Kantor *et al.* (2018 ▸). In that work, an analysis of a system’s achromaticity was performed based on the measurement of its linear spatial resolution, yielding useful information on the wavelength range for temperature fitting and other aspects such as minimum hotspot size and effect of numerical aperture (NA). Here we present an alternative method for quantifying the aberrations and justifying the choice of the optics and spectral range. The system of the geoHEAT lens in combination with a simple BK7 lens of different focal lengths was simulated using the ray-tracing program *ZEMAX*. We studied also different catalog achromats to see the influence of various types of optics on the system performance and to understand why certain approaches would work (such as reducing the front aperture of the first lens) and in which cases they would be unnecessary. Simulations of our system were carried out using a *ZEMAX* black box design of the geoHEAT lens provided by the manufacturer and lens data of a simple BK7 lens (Thorlabs, *f* = 1000 mm). The simulations show that this system produces about 12 mm of focal shift over the spectral range 600–900 nm [Fig. 4 ▸(*a*)]. The curve of the focal shift dependence on the wavelength shows that by omitting the 600–640 nm spectral range one could, in principle, reduce the chromatic aberrations by more than 50%.

However, another type of aberration – the spherical aberrations caused by light rays being refracted unevenly along the curvature of the spherical surface of the lens – appears to be more significant than the chromatic ones. The *ZEMAX* simulations show that in the spectral range 640–850 nm (which is our preferred spectral range) the longitudinal spherical aberration considering the full 19 mm lens aperture of the geoHEAT (NA = 0.16) is about 12 mm [Fig. 4 ▸(*b*)]. The longitudinal spherical aberration (LSA) increases with increasing image magnification of the system. For example, for the long focal lenses with *f* = 750 mm (magnification 12.5) and 1000 mm (magnification 16.6), the LSA increases from 6 mm (0.8% of the focal length) to about 12 mm (1.2% of the focal length), respectively. Therefore a compromise between the system magnification and an acceptable LSA needs to be found.

This can be accomplished with the help of simulation of a geometric encircled energy. This quantity gives a fraction of light at the focal point focused to a certain width of aperture (*e.g.* a spectrometer entrance slit). For a system with large aberrations this fraction is much less than 1 due to the focus blur caused by the large variation of the foci position along the beam axis.

**Figure 5:**
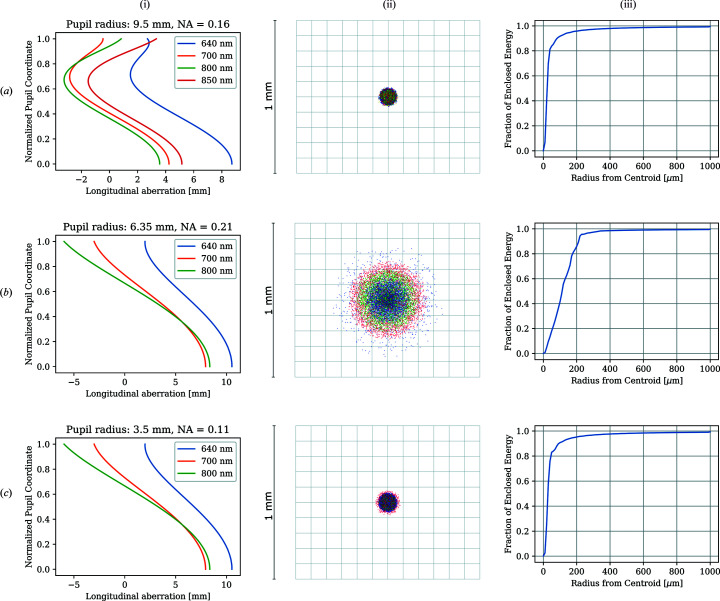
Longitudinal aberrations (i), image simulation of a 5 µm diameter circle (ii) and encircled energy (iii) for (*a*) the geoHEAT system at ECB, (*b*) the Thorlabs lens system used by Giampaoli *et al.* (2018 ▸; (*c*) the same Thorlabs lens system with a front aperture of 7 mm.

In Fig. 5 ▸ we are comparing longitudinal aberration, image quality and the geometric encircled energy for various optical systems. For the geoHEAT lens paired with the *f* = 1 m BK7 lens, 95% of energy for all considered wavelengths (640–850 nm) focus into a 200 µm slit opening [Fig. 5 ▸(*a*)(iii)]. For comparison, a lens system such as that used by Giampaoli *et al.* (2018 ▸) (Thorlabs, AC127-030-B, *f* = 30.0 mm, 1/2′, NA = 0.21; and AC254-500-B, *f* = 500.0 mm, 1′) produces an 80 mm spread of focus along the optical axis (16% of the distance between the second lens and image) caused primarily by spherical (longitudinal) aberrations [Fig. 5 ▸(*b*)(i)]. In such a system, shorter wavelengths (650 nm) are more present in the 200 µm aperture than the higher wavelengths, which would consequently bias the slope of the Planck fit towards lower temperatures. It is a common practice to place an aperture in front of the first achromat to decrease aberrations (Mezouar *et al.*, 2017 ▸). This method improves the performance of the optical system because the spherical aberrations are reduced, not the chromatic ones as commonly stated. Fig. 5 ▸(*b*)(i) shows that the rays significantly deviate from 0 at the outer 30% of the lens aperture. When this part of the lens is cut off (by the aperture), the aberration becomes much smaller and so the temperature measurement more accurate [Fig. 5 ▸(*c*)(i)]. However, reducing the NA is not a universal fix for all systems and may not be applicable to specialized optics such as geoHEAT or MITUTOYO objectives as they are designed in a way that all the rays going through the entire aperture exhibit little variation of the focus displacement along the optical axis. For the Thorlabs achromat, a smaller aperture of 7 mm diameter (NA = 0.11) improves the overall performance of the system: >95% of the energy in the focal point is now focused down to a 200 µm radius and the overall aberration is reduced to 16 mm [Fig. 5 ▸(*c*)(i)]. Hence, the performance of the reduced-aperture Thorlabs system and the full-aperture geoHEAT systems in terms of optical aberrations become comparable.

The disadvantage of the aperture approach is the decreased amount of collected light through a smaller aperture of the lens (note that a reduction of the Thorlabs NA by a factor of two is necessary to yield a satisfactory optical system) and so loss in spatial resolution.

To summarize, placing an aperture in front of the geoHEAT does not improve the already good performance any further; hence we take advantage of collecting light through the entire diameter of the lens (19 mm diameter, NA = 0.16). Placing a small aperture instead of the spectrometer entrance slit without knowing the magnitude of the longitudinal spherical aberration of the system is not recommended as it may alter the ratio of intensities of different wavelengths reaching the detector and hence affect the temperature measurement. However, many transmission optics elements, unavoidable in such system, such as dichroic mirrors (introducing the laser light into the pyrometry path) or beamsplitters (for imaging), do in fact modulate the relative intensities reaching the spectrometer but these are corrected for by the calibration procedure. Any deviations from the ideal black-body radiation are thus taken into account, including the aberrations.

### 1.5. Online pressure measurement

A ruby system for *in* *situ* pressure measurements is implemented on the same optical table as the laser heating setup. A Melles Griot laser (85-BLS-601) with 457.9 nm wavelength is used to excite fluorescence of a ruby crystal. The ruby R1 emission line is detected by a Princeton Instrument spectrometer (Acton Spectra Pro 2300i) and camera (PiXIS 100 detector) system. The ruby measurement is carried out from the downstream side of the sample by moving in a dichroic mirror (DM in Fig. 1 ▸) for laser beam reflection and a mirror (M5 in Fig. 1 ▸) which sends the fluorescence signal to the ruby detection system.

## 2. Pulsed-laser heating techniques

**Figure 6:**
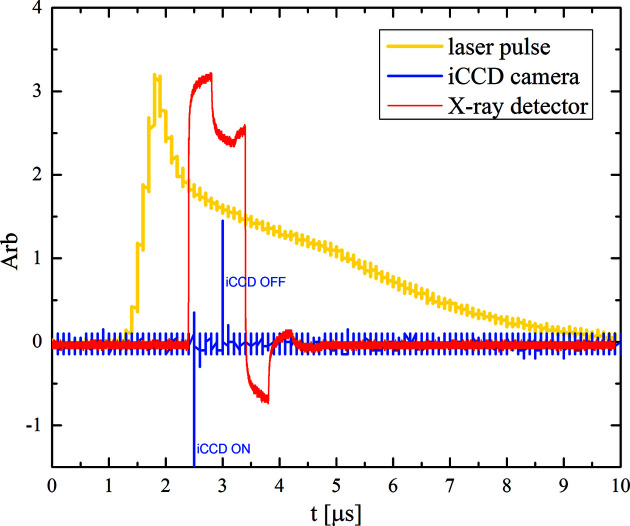
Example of oscilloscope traces of a laser pulse shape (yellow) and detection windows of the X-ray detector (red) and iCCD camera (blue).

A pulsed-laser heating technique has been developed at ECB using two approaches: (1) integrated short laser pulses of a couple of microseconds at a kHz repetition rate are combined with gated temperature measurement and X-ray diffraction (XRD) acquisition, and (2) a single laser pulse of milliseconds duration with simultaneous temperature and diffraction collection is executed in a single shot. For this purpose, the *on-axis* fiber laser is modulated by a digital delay/pulse generator (DDG) producing laser pulses as short as a few microseconds (Fig. 6 ▸). The DDG triggers the laser, iStar camera, X-ray detector and/or X-ray shutter, and controls their exposure times and relative delays. An example of the laser trace and timing of the devices is presented in Fig. 6 ▸.

### 2.1. High repetition heating with short pulses

**Figure 7:**
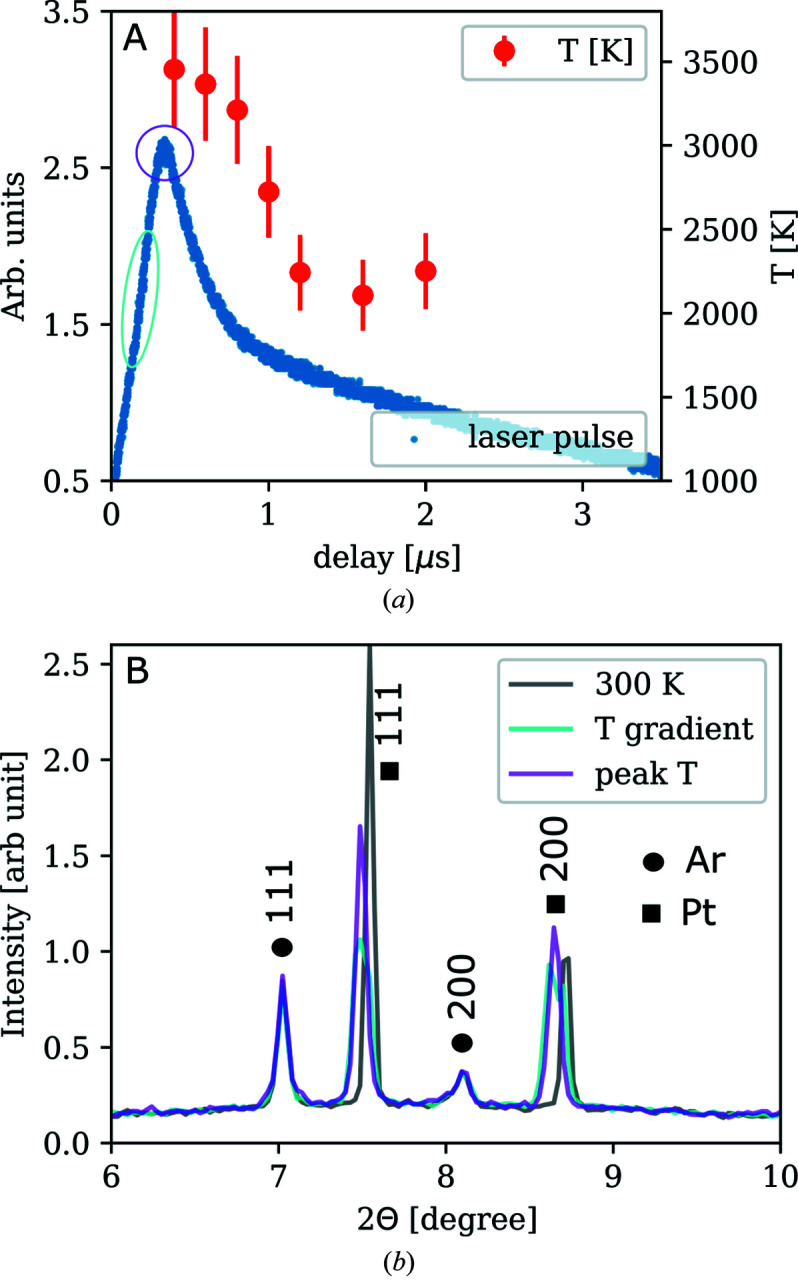
(*a*) Laser pulse intensity (left axis) and temperature (right axis) versus time delay. (*b*) Diffraction patterns of the cold sample (black); of the sample subjected to steep temporal temperature gradient (cyan) causing broadening/splitting of the diffraction peaks; and of the sample at the peak temperature (violet). The XRD pattern is a result of 80000 shots.

A Pilatus Si 1M X-ray detector has been used for diffraction measurements during heating with the laser pulses. For example, in a proof-of-principle experiment to study the melting of metals such as Fe and Pt, laser pulses of 1 µs with a 4 µs-long tail were applied to the sample at a 10 kHz repetition rate (Goncharov *et al.*, 2010 ▸). By changing the delay of the Pilatus exposure window with respect to the arrival of the laser pulse one can monitor the lattice expansion at different temperatures along the laser pulse intensity profile. The emission from the hot sample is measured by the iStar camera with the same acquisition time window as the Pilatus 1M detector. To collect adequate diffraction intensities on Fe and Pt, it was necessary to integrate tens of thousands of shots into one image. Fig. 7 ▸ shows examples of XRD patterns of Pt and Ar at 30 GPa at ambient and temporally elevated temperatures. Expansion of the lattice during laser pulses is manifested by the diffraction peaks shifting towards the lower 2θ angles. While this technique has a great potential when studying reversible processes, detecting phase transitions such as melting in metals suffers from the shot-to-shot non-reproducibility most likely due to surface changes upon melting.

### 2.2. Single-shot heating

**Figure 8:**
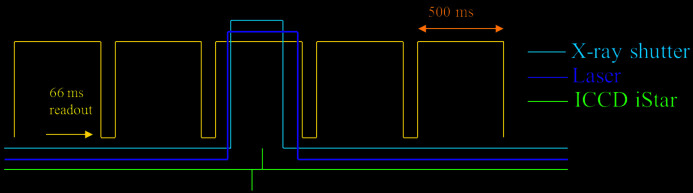
The PerkinElmer XRD 1621 (yellow) running in a silent free running mode. An external trigger signal picks the next rising edge of the detector readout signal from the frame grabber card. The activated rising edge triggers a digital delay generator (DDG), which in turn triggers the X-ray shutter (blue), laser (purple) and iCCD camera (green) with the possibility to vary delays and widths of the exposure windows.

To minimize carbon contamination from diamonds or reactions with pressure-transmitting media, it is desirable to keep the heating time at a minimum, *i.e.* only as long as sufficient X-ray diffraction and emission intensities are collected in a single exposure. A large-area flat-panel detector with a fast read-out time (67 ms at its highest repetition rate of 15 frames s^−1^), the PerkinElmer XRD 1621 detector, was employed to secure the most optimal performance at hard X-ray energies. A customized electronic device was used to synchronize the readout period of the detector with the X-ray shutter, heating lasers and the iStar camera (Fig. 8 ▸) to collect all the measurement data in a single acquisition.

Single-shot laser heating experiments with exposure times of typically a couple of hundred milliseconds were carried out to study melting of iron at high pressures. Knowledge of the melting curve of iron has a well known implication for constraining temperatures in the Earth’s core (Boehler, 1993 ▸; Sinmyo *et al.*, 2019 ▸, and references therein). However, there are large differences in reported melting temperatures due to experimental challenges such as carbon contamination of iron samples from the diamond anvils or reaching high enough temperatures at pressures above 1 Mbar (Morard *et al.*, 2018 ▸; Aprilis *et al.*, 2019 ▸).

We applied the single-shot technique to an iron sample (foil with starting thickness no more than 3 µm) compressed with a salt (NaCl) pressure-transmitting medium in a DAC to pressures of up to 190 GPa (pressure derived at room temperature before heating). XRD patterns were collected during 200–500 ms laser pulses. Depending on the temperature reached, the length of the emission detection window was adjusted to yield optimal intensities. The X-ray illumination of the sample was timed with the laser pulse and the PerkinElmer detector using a fast X-ray shutter (Fig. 8 ▸). After each acquisition at high temperature, diffraction data of the quenched sample were collected by repeating the acquisition without enabling the laser. Subsequent data were acquired at increasing laser power and thus higher temperatures.

**Figure 9:**
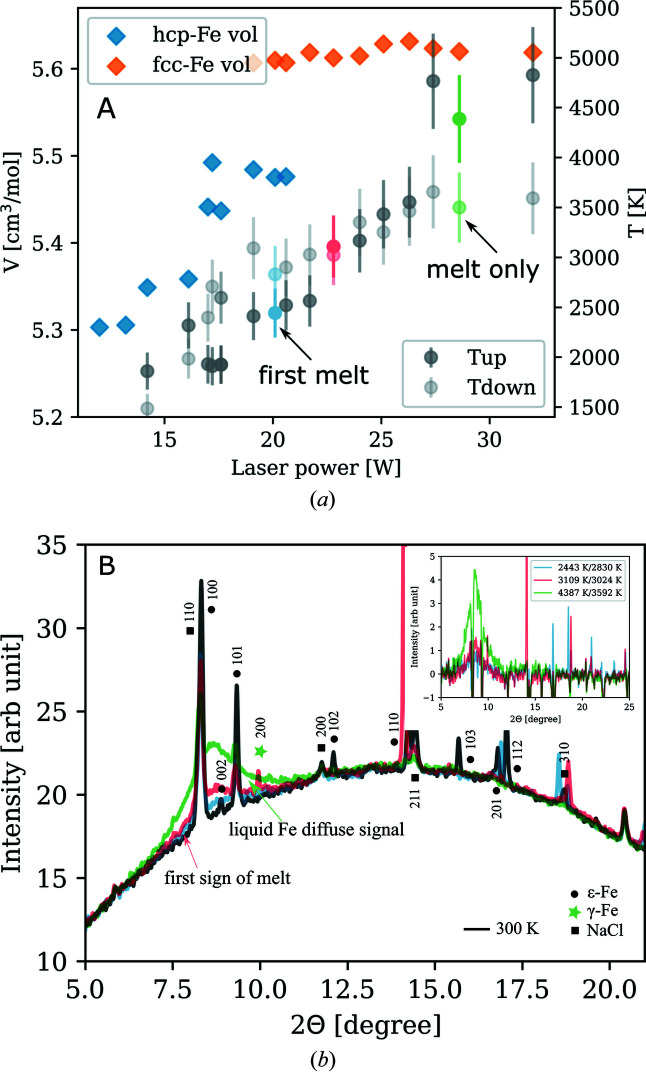
(*a*) Unit-cell volume (left axis) of ɛ- and γ-Fe and temperature (right axis) as a function of laser power, at 70 GPa. (*b*) XRD patterns of the sample assembly before heating (black), at temperatures of the first sign of melting (red) and when the iron sample is almost completely molten (green) at temperatures above the melting curve. The inset shows the residual diffuse signal after subtracting the room-temperature pattern.

Unit-cell volumes of h.c.p.-Fe (ɛ-Fe) and f.c.c.-Fe (γ-Fe) have been measured as a function of laser power and temperature. Fig. 9 ▸ shows an example of such data collection at 70 GPa [pressure calculated from the equations of state of Fe (Dewaele *et al.*, 2006 ▸) and NaCl (Sata *et al.*, 2002 ▸) before heating]. The temperatures are derived from the emission signal for each side of the sample separately (denoted as ‘Tup’ and ‘Tdown’). In previous studies, the onset of melting has been identified by the observation of the first rings of diffuse scattering and a plateau in the lattice volume versus laser power relationship (Anzellini *et al.*, 2013 ▸). In our data at 70 GPa, for example, this would lead to a melting temperature of iron at 3000 ± 300 K (Fig. 9 ▸, red). The temperature error is estimated as 10% of the temperature value. Error estimation broadly agrees well with the sliding two-color pyrometry analysis presented by Morard *et al.* (2018 ▸). The factors that contribute to the temperature errors are axial/radial temperature gradients (discussed below), fast changes in optical properties rising from sample re-crystallization that is being integrated over the exposure time of the pyrometry measurements, and the combination of the detecting system sensitivity and the signal level (signal-to-noise ratio). All these aspects may vary from shot-to-shot measurement and may be difficult to control especially when the sample behavior under the irradiation is dynamic. However, we believe the 10% error bar is a good representation of the uncertainties of the collected temperature data.

**Figure 10:**
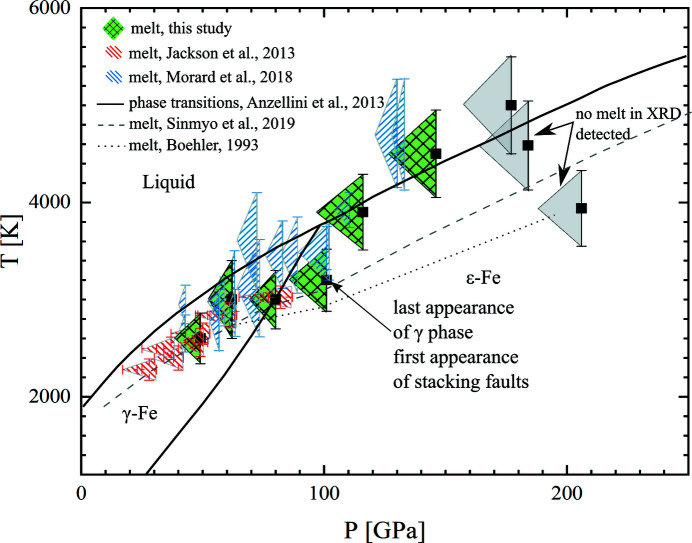
Melting points from our study (green symbols with grid) compared with the previous studies of Jackson *et al.* (2013 ▸) (red), Anzellini *et al.* (2013 ▸) (solid curves), Morard *et al.* (2018 ▸) (blue), Boehler (1993 ▸) and Sinmyo *et al.* (2019 ▸). Our uncertainty in the melting point is constrained by the triangles – the left corners are the starting pressure at room temperature and the black squares are pressures after accounting for the additional thermal pressure (Dewaele *et al.*, 2006 ▸). We applied this representation also to the data of Jackson *et al.* (2013 ▸) and Morard *et al.* (2018 ▸) to emphasize the variation in the assumed thermal pressure by different authors. Plain symbols in our data mean no melting was detected. Stacking faults were detected at pressures above 100 GPa. The temperature error is estimated as 10% of the temperature value.

The results are summarized in Fig. 10 ▸. Each data point marks the starting pressure before heating (left corner of a triangle) and the final pressure at high temperature calculated from the thermal equation of state (Dewaele *et al.*, 2006 ▸) (black squares). We applied such a representation to the data of Jackson *et al.* (2013 ▸) and Morard *et al.* (2018 ▸) to stress the differences in the assumed thermal pressures. Within all the uncertainties in pressures and temperatures, below the triple point, our results agree better with the study of Jackson *et al.* (2013 ▸), Morard *et al.* (2018 ▸) and Sinmyo *et al.* (2019 ▸) but are systematically lower than the melting curve of Anzellini *et al.* (2013 ▸).

At pressures above the γ-liquid triple point where no γ-phase reflections were detected from the solid phase, we observe higher melting temperatures of the ɛ-Fe phase than those of the γ-phase. At pressure above 100 GPa, our data favor higher melting temperatures than those of Boehler (1993 ▸) and Sinmyo *et al.* (2019 ▸), for example. At pressures close to 2 Mbar, no significant diffuse scattering was detected in the X-ray diffraction patterns. It is possible that the melting indeed occurred but, due to the very thin sample at those pressures, the X-ray photon number in such a short time period may be insufficient to detect significant diffuse scattering that would hint at the presence of melt.

**Figure 11:**
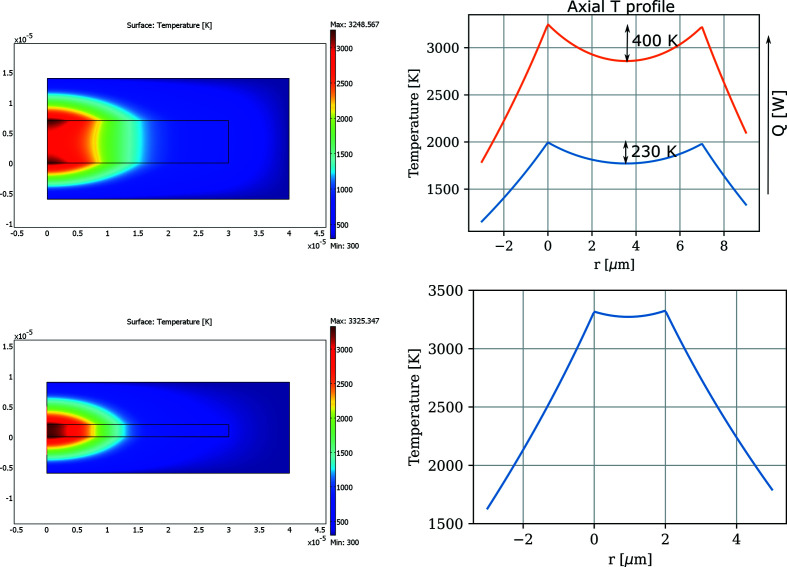
Temperature gradients through the Fe sample, 7 µm (top) and 2 µm (bottom) thick, respectively, sandwiched between layers of salt pressure medium. The 2D plots on the left show axisymmetric models with the symmetry axis defined by the laser beam axes. The temperature profiles plots on the right are calculated along the symmetry axis. While the 7 µm thick sample sustains ∼200 K axial temperature gradient at 2000 K, at 3000 K the gradient increases to 400 K. The FEM calculations were performed with a sample thermal conductivity of 40 W m^−1^ K^−1^ (Konôpková *et al.*, 2016 ▸).

As pointed out by Anzellini *et al.* (2013 ▸), thicker samples (>3 µm) may exhibit large temperature gradients at high temperatures. We performed a finite-element method (FEM) analysis of a continuously double-side laser heated sample in a DAC of various thicknesses to estimate the axial temperature distribution in the sample. The thermal conductivity of the sample was assumed to be 40 W m^−1^ K^−1^ (Konôpková *et al.*, 2016 ▸) and the laser spot size 20 µm FWHM. The laser power, *Q*, was adjusted to reach different temperatures on the surface of the sample. With 7 µm-thick samples, the axial temperature gradients may reach up to 400 K (Fig. 11 ▸, top) at about 3000 K measured on the surface of the foil. This problem would be mitigated using thinner (2 µm) samples also at lower pressures where the axial temperature gradient becomes negligible (Fig. 11 ▸, bottom).

**Figure 12:**
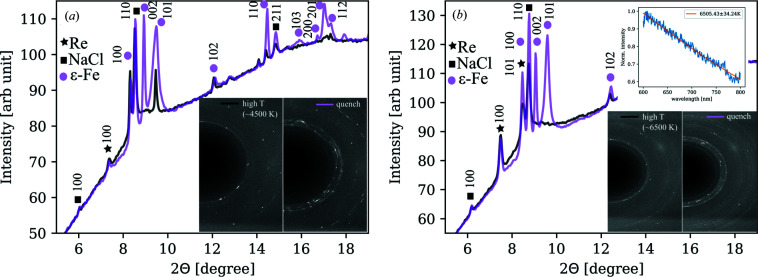
Diffraction patterns at (*a*) 96 GPa and (*b*) 126 GPa. The resulting quenched material has a strong, almost continuous, 002 reflection and a broad, asymmetric, 101 ɛ-Fe peak.

The quenched material from almost completely molten h.c.p.-Fe shows diffraction patterns with streaks and diffuse 101 peak (Fig. 12 ▸), which has not been observed at lower pressures, in the f.c.c.-Fe stability field. Such signatures in the XRD patterns are typically attributed to the presence of stacking faults in the structure; in this case the disorder is related to the f.c.c.–h.c.p. martensitic transformation. The stacking disorder was shown to play a major role in the f.c.c.-to-h.c.p. transition of Xe (Cynn *et al.*, 2001 ▸), in Co at high temperatures (Frey & Boysen, 1981 ▸) and at high pressures (Yoo *et al.*, 2000 ▸). In a diffraction pattern of an h.c.p. crystal, stacking faults are manifested by strong and sharp reflections *hkl* with *h* − *k* = 3*n* and *l* = 2*n* (*n* is an integer) while others are more diffuse (Frey & Boysen, 1981 ▸). The extent to which these disorders occur depends heavily on thermal history, grain size and the actual pressure/temperature conditions (Frey & Boysen, 1981 ▸). In iron, stacking faults were proposed to exist at conditions of the Earth’s core but were not observed in the experiments of a temperature quenched iron sample at 160 GPa (Mikhaylushkin *et al.*, 2007 ▸). This may suggest that the formation of the stacking faults is activated upon rapid cooling from completely molten h.c.p.-Fe. This corroborates the fact that our observation of the diffuse streaks in the quenched patterns coincides with a recent experiment of Hrubiak *et al.* (2018 ▸), which also used flash heating with laser pulses of 1–5 ms duration. In their experiments, at pressures above 95 GPa and above a certain temperature ‘threshold’, a microstructure begins to appear in the quenched Fe samples producing XRD patterns similar to the ones presented in Fig. 12 ▸.

## 3. Conclusion

Within this work we have described a laser heating system developed at the Extreme Conditions Beamline of PETRA III synchrotron, DESY, for high-pressure high-temperature studies using diamond anvil cells. The optics layout is described in detail and has been simulated by the ray-tracing program * ZEMAX* to study the effects of aberrations. We find that the spherical (longitudinal) aberrations, as opposed to the chromatic aberrations, are dominant in the optical setups commonly used for DAC laser heating applications with refractive optics. The geoHEAT system is shown to have a comparable performance with the reduced-aperture Thorlabs system in terms of aberrations while maintaining larger numerical aperture. Standard cw heating of the sample under pressures has been extended to pulsed-laser heating to enable shorter heating duration to minimize reactions of the sample. As an example of the capability of the pulsed laser heating system we revisit the melting curve of iron up to 2 Mbar.
